# Sibling conflict during COVID‐19 in families with special educational needs and disabilities

**DOI:** 10.1111/bjep.12451

**Published:** 2021-08-22

**Authors:** Umar Toseeb

**Affiliations:** ^1^ Department of Education University of York UK

**Keywords:** bullying, COVID‐19, disability, family, sibling conflict, special education

## Abstract

Young people with special educational needs and disabilities (SENDs) and their families have been particularly hard hit by the COVID‐19 pandemic. In this longitudinal study, sibling conflict in these families during and after the first lockdown in the United Kingdom was investigated. Online questionnaires were completed by 504 parents of young people with SENDs at four time points between 23 March 2020 and 10 October 2020 (over half completed the questionnaire at multiple time points). As lockdown progressed, young people with SENDs were more likely to be picked on or hurt by their siblings compared with earlier stages of the lockdown but there was no change in how frequently they harmed or picked on their siblings. After lockdown, both perpetration and victimization decreased but not to the same rates as the first month of lockdown. Young people with SENDs with severe or complex needs were somewhat protected from sibling conflict. Findings are discussed with reference to implications for support and planning for future pandemics.

## Background

Siblings are an important part of children’s lives. They can have a positive effect on children’s development, but not all interactions with siblings are positive. Some are characterized by conflict and bullying. Indeed, nearly half of children report being involved in sibling bullying; a form of persistent sibling conflict (Toseeb, McChesney, & Wolke, 2018). School closures, as a result of the first COVID‐19 lockdown in the United Kingdom (UK), starting on 23 March 2020, meant that most children were spending almost all of their time at home with their siblings. Such prolonged confinement with siblings may have led to increased time and opportunity for sibling conflict. Therefore, the aim of this study was to investigate sibling conflict during and after the first COVID‐19 lockdown in the United Kingdom in families with special educational needs and disabilities (SENDs).

### Special educational needs and disabilities

Young people with SENDs have impairments in functioning which might affect their ability to learn. These include impairments in communication and interaction (e.g., autism spectrum conditions), cognition and learning impairments (e.g., dyslexia), sensory and physical disabilities (e.g., visual impairments), and or social, emotional, and mental health difficulties (e.g., attention deficit hyperactivity disorder). Approximately 15% of all school‐aged children have a SEND (DfE, 2021). Of these, approximately 20% have an education, health, and care plan (EHCP). An EHCP is a legal document that sets outs the educational, health, and social care needs of a young person with SENDs. In general, all children with an EHCP have a SEND but only those whose additional needs cannot be met by existing SEND support have an EHCP. Preliminary evidence suggests that young people with SENDs have been particularly hard hit during the COVID‐19 pandemic (Asbury, Fox, Deniz, Code, & Toseeb, 2021; Toseeb, Asbury, Code, Fox, & Deniz, 2020).

Young people with SENDs often have complex needs and rely on carefully established routines and support networks which, if disrupted, can lead to excessive distress (American Psychiatric Association, 2013). For example, many children with SENDs are likely to access speech and language therapy, occupational therapy, mental health support, or attend specialist school provision. Such support networks were abruptly broken during the first lockdown in the United Kingdom. Parents were suddenly required to care for and educate their children all day every day without their usual support systems, leading to a number of unmet needs in these families (Toseeb, Asbury, et al., 2020). Young people with SENDs and their parent carers experienced high levels of psychological distress during the first UK lockdown (Asbury et al., 2021). An accumulation of such psychological distress is a risk factor for intra‐familial conflict. For example, harsh parenting increased during the COVID‐19 pandemic, which was likely a result of increased stressors experienced by parents (Lee & Ward, 2020). It seems pertinent, therefore, to investigate sibling conflict in young people with SENDs during the first COVID‐19 lockdown, when routines and support networks were abruptly disrupted, and after, when they were beginning to be re‐established.

### Sibling conflict

The term sibling conflict is used here to refer to negative physical, social, and psychological interactions between siblings and, in its most persistent form, includes sibling bullying. Estimates based on population studies suggest that half of all children report being involved in persistent sibling conflict (Toseeb et al., 2018). This decreases to a third by the time they reach early adolescence (Toseeb, McChesney, Oldfield, & Wolke, 2020). Persistent sibling conflict is associated with poor mental health in the short‐term (Bowes, Wolke, Joinson, Lereya, & Lewis, 2014; Liu et al., 2020; Lopes, Relva, & Fernandes, 2019; Tucker, Finkelhor, Turner, & Shattuck, 2013; van Berkel, Tucker, & Finkelhor, 2018) and long‐term (Dantchev, Hickman, Heron, Zammit, & Wolke, 2019; Dantchev & Wolke, 2018; Dantchev, Zammit, & Wolke, 2018). Therefore, persistent sibling conflict is a public health concern requiring the attention of parents, policy makers, and practitioners.

A number of structural family and parenting characteristics may explain sibling conflict. The resource control theory suggests that asymmetries amongst social groups (or in this instance, family systems) foster social dominance (Hawley, 1999). That is, differential access to finite parental resources (e.g., affection, attention, and material goods) may lead to sibling conflict (Tanskanen, Danielsbacka, Jokela, & Rotkirch, 2017). Such dominance of parental resources is influenced by structural family characteristics. As the number of siblings in the household increases, parental resources are spread more thinly and so the frequency of persistent sibling conflict may also increase (Toseeb, McChesney, Dantchev, & Wolke, 2020). Similarly, first‐born children are likely to experience a loss in parental resources when a new sibling enters the household and therefore are more likely to be the perpetrators of siblings conflict (Toseeb, McChesney, Dantchev, et al., 2020). Parenting and parental characteristics may also influence the levels of persistent sibling conflict. Harsh parenting is associated with higher levels of persistent sibling conflict (Toseeb, McChesney, Dantchev, et al., 2020). This may be because children are socialized by modelling parents’ behaviours (Bandura, 1977) and they use parent‐child interactions as templates for interactions with their siblings (Bowlby, 1969). But such structural family and parenting characteristics are unlikely to operate in isolation.

Child‐level individual differences are also important predictors of persistent sibling conflict. Young people’s individual characteristics may evoke a reaction from others in their environment or they may seek out situations that are congruent with their innate propensities (Plomin, 2018). The literature suggests that boys are more likely to be involved in persistent sibling conflict than girls (Tucker, Finkelhor, Shattuck, & Turner, 2013). In addition, those with pre‐existing mental health difficulties, low self‐esteem, or social difficulties are also more likely to be involved in persistent sibling conflict (Dantchev & Wolke, 2019; Phillips, Bowie, Wan, & Yukevich, 2016). These child‐level individual differences are likely to interact with family‐, community‐, and society‐level factors to influence persistent sibling conflict. Such a conceptualization is congruent with Bronfenbrenner’s ecological systems perspective whereby children’s development takes place within the context of wider ecosystems (Bronfenbrenner, 1979). One key child‐level individual difference which is likely to influence sibling conflict is the presence of a SEND.

### Sibling conflict and special educational needs and disabilities

There are a number of reasons why sibling conflict may be higher in families in which one or more child has SENDs. Social and communication difficulties may make children with SENDs more prone to being picked on by siblings, as is the case for conflict with peers (Cappadocia, Weiss, & Pepler, 2012). Neurotypical siblings of young people with SENDs may also have some social impairments, such as not being able to respond appropriately in social situations (Constantino et al., 2006), which may increase the risk of escalation of sibling conflict. Parents of young people with SENDs may experience higher levels of psychological distress compared with parents of neurotypical young people (Hoffman, Sweeney, Hodge, Lopez‐Wagner, & Looney, 2009), thus increasing the risk of intra‐familial conflict (Lee & Ward, 2020). Additionally, young people with SENDs may require disproportionate time, attention, and support from parents fuelling competitive behaviour and aggression amongst siblings (Felson, 1983). Perceived parental favouritism in these families is associated with negative sibling relationships (McHale, Sloan, & Simeonsson, 1986). Indeed, from an evolutionary perspective siblings are competitors for parental attention, affection, and material goods (Tanskanen et al., 2017) and young people with SENDs might represent particularly tough competition in some families.

These predictions appear to be confirmed by previous research. In a UK‐based population study of nearly 14,000 children (475 with autism spectrum conditions), those with autism spectrum conditions were more likely to report being involved in persistent sibling conflict, both as victims and perpetrators (Toseeb et al., 2018). A follow‐up study of the same young people during adolescence confirmed the increased risk of persistent sibling conflict for those with autism spectrum conditions (Toseeb, McChesney, Oldfield, et al., 2020). Therefore, persistent sibling conflict is an area of concern in young people with a specific type of SEND, even in normal times.

### The current study

The current study investigated sibling conflict during and after the first COVID‐19 lockdown in the United Kingdom in families where one child had a SEND. The COVID‐19 lockdowns differed slightly across different regions of the United Kingdom as devolved governments of Northern Ireland, Scotland, and Wales made some local decisions. All four nations of the UK went into lockdown on the 23 March 2020. Broadly, similar restrictions were in place across the four nations although there were some local variations. The first lockdown in the United Kingdom presented a new and potentially stressful situation for SEND families. A withdrawal of the usual support networks across all nations of the United Kingdom may have led to high levels of stress in families, who were already struggling pre‐pandemic. The increased time siblings were spending together meant that there was more opportunity for conflict amongst them. To the best of the author’s knowledge, this was the first study to investigate sibling conflict in these families during the COVID‐19 pandemic. Additionally, much of the previous work on persistent sibling conflict in SEND families comes from a single UK cohort using self‐report data. This may be problematic because individuals with SENDs (e.g., those with autism spectrum conditions) may not recognize harmful behaviours (Frith & Hill, 2004). Finally, much of the previous work has focussed on young people with autism spectrum conditions without investigating the effect of co‐occurring conditions. This is problematic because SENDs tend to co‐occur, meaning that a young person with one type of SEND is at increased risk of another type of SEND. Investigating SENDs in isolation is unlikely to provide information about the specific area of difficulty that is associated with sibling conflict. This study, therefore, addressed three research questions:

1. What were the rates of sibling conflict in families with SENDs during and after the first lockdown in the United Kingdom? (Research Question 1)
2. Did the rates of sibling conflict in families with SENDs change from the first month of lockdown until after schools fully reopened for face‐to‐face teaching six months later? (Research Question 2)
3. Which factors predicted sibling conflict in families with SENDs during and after the first lockdown in the United Kingdom? (Research Question 3)

## Methods

### Ethics

The study was approved by the Education Ethics Committee at the University of York (Reference: 20/05). Parents of young people with SENDs provided informed consent.

### Participants and design

There were 504 parent carers of young people with SENDs who took part in an online questionnaire. Those with more than one child with SEND were asked to focus on one child. Parents were recruited via existing research networks (e.g., Autistica and the National Autistic Society), non‐mainstream schools (e.g., special schools, pupil referral units), online platforms (e.g., Twitter, Facebook groups ), and a paid research site (i.e., Prolific).

The study design was intended to be a longitudinal cohort study but it turned out to be quite complex. Parents took part at *one or more* of four time points: 23 March 2020–22 April 2020 (Time 1, T1), 23 April 2020–22 May 2020 (Time 2, T2), 23 May 2020–22 June 2020 (Time 3, T3), and 29 September 2020–10 October 2020 (Time 4, T4). At each time point, all parents from the previous time points were invited to take part in the follow‐up questionnaire. Given the level of sample attrition, new participants were recruited at each time point to boost the sample size and maximize power (see Table 1). Sample attrition was calculated as the proportion of participants who took part in all previous time points. The sample attrition was as follows: T2 – 55% (171 parents took part at T1 but not T2), T3 – 73% (297 parents took part at T1 or T2 but not T3), and T4 – 69% (330 parents took part at T1, 2, or 3 but not T4). There were 249 (49%) participants who only took part at one time point, 158 (31%) who took part at two time points, 51 (10%) who took part at three time points, and 46 (9%) who took part at all four time points.

**Table 1:** Sample demographics

	Overall	Time 1	Time 2	Time 3	Time 4
**Sample size**	**504**	**312 (100%)**	**238 (100%)**	**178 (100%)**	**174 (100%)**
Follow‐up^a^	–	0	141 (59%)	112 (63%)	145 (83%)
New	–	312 (100%)	97 (41%)	66 (27%)	29 (17%)
**Respondent**	**504 (100%)**	**311 (100%)**	**231 (100%)**	**167 (100%)**	**174 (100%)**
Mother	464 (92%)	291 (94%)	217 (94%)	148 (89%)	154 (88%)
Father	30 (6%)	13 (4%)	8 (3%)	16 (9%)	17 (10%)
Other (foster, adoptive, grandparents etc.)	10 (2%)	7 (2%)	6 (2%)	3 (2%)	3 (2%)
**Country within United Kingdom**	**494 (100%)**	**311 (100%)**	**237 (100%)**	**171 (100%)**	**170 (100%)**
England	463 (94%)	296 (95%)	227 (96%)	163 (95%)	156 (92%)
Northern Ireland	17 (3%)	8 (2%)	0 (0%)	5 (3%)	7 (4%)
Scotland	10 (2%)	5 (2%)	6 (2%)	2 (1%)	4 (2%)
Wales	4 (1%)	2 (1%)	4 (2%)	1 (1%)	3 (2%)
**Household Income**	**504 (100%)**	**305 (100%)**	**232 (100%)**	**168 (100%)**	**168 (100%)**
Below median income	252 (50%)	156 (51%)	112 (48%)	79 (47%)	66 (40%)
Above median income	252 (50%)	149 (49%)	120 (52%)	89 (53%)	102 (61%)
**Young Person Age (Years)**	**10.54 (3.55)**	**9.90 (3.46)**	**10.96 (3.58)**	**10.54 (3.54)**	**11.07 (3.51)**
**Young Person Sex**	**493 (100%)**	**311 (100%)**	**235 (100%)**	**170 (100%)**	**170 (100%)**
Girl	147 (30%)	93 (30%)	65 (28%)	59 (35%)	51 (30%)
Boy	346 (70%)	218 (70%)	170 (72%)	111 (65%)	(70%)
**Young Person Ethnicity**	**495 (100%)**	**311 (100%)**	**238 (100%)**	**171 (100%)**	**171 (100%)**
White British	443 (89%)	283 (91%)	215 (90%)	156 (91%)	155 (91%)
Ethnic minority	52 (11%)	28 (9%)	23 (10%)	15 (9%)	16 (9%)
**Number of Siblings**	**496 (100%)**	**312 (100%)**	**238 (100%)**	**171 (100%)**	**171 (100%)**
One	270 (55%)	166 (53%)	125 (53%)	96 (56%)	102 (60%)
Two	121 (24%)	76 (24%)	60 (25%)	39 (23%)	36 (21%)
Three	69 (14%)	40 (13%)	34 (14%)	20 (12%)	20 (12%)
Four or more	36 (7%)	30 (10%)	19 (8%)	16 (9%)	13 (7%)
**Young Person First Born**	**495 (100%)**	**311 (100%)**	**238 (100%)**	**171 (100%)**	**171 (100%)**
No	303 (61%)	181 (58%)	142 (60%)	104 (61%)	114 (67%)
Yes	192 (39%)	130 (42%)	96 (40%)	67 (39%)	57 (33%)
**Young Person Verbal Ability**	**504 (100%)**	**311 (100%)**	**235 (100%)**	**172 (100%)**	**174 (100%)**
Verbal	423 (84%)	257 (83%)	197 (84%)	144 (84%)	153 (88%)
Minimally verbal	81 (16%)	54 (17%)	38 (16%)	16 (28%)	21 (12%)
**Young Person Educational Placement**	**496 (100%)**	**312 (100%)**	**238 (100%)**	**171 (100%)**	**171 (100%)**
Mainstream	247 (50%)	127 (41%)	114 (48%)	84 (49%)	96 (56%)
Non‐mainstream	249 (50%)	185 (59%)	124 (52%)	87 (51%)	75 (44%)
**Young Person Education Health and Care Plan**	**496 (100%)**	**312 (100%)**	**238 (100%)**	**171 (100%)**	**171 (100%)**
No	170 (34%)	93 (30%)	74 (31%)	56 (33%)	64 (37%)
Yes	326 (66%)	219 (70%)	164 (69%)	115 (67%)	107 (63%)

Note

Values represent maximum sample size. Some Ns differ to other tables due to missing data on specific measures. The % in bold are a function of the sub‐headings below and not of the overall sample size.

^a^ Parents who took part in at least one of the previous time points

^b^ Approximate median income in the United Kingdom (pre‐income tax).

### Measures

#### Demographic information

Parents were asked a number of demographic questions. All questions relating to the young person refer to the young person with SEND (i.e., not the sibling).

#### Relationship to the young person

Parent carers were asked ‘What is your relationship to your child?’ and responded by selecting one of three options (0 = *Mother*, 1 = *Father*, 2 = *Other*).

#### Country within the United Kingdom

Parent carers were asked ‘Which part of the United Kingdom do you live in?’ and responded by selecting one of four options (0 = *England*, 1 = *Scotland*, 2 = *Northern Ireland*, 3 = *Wales*).

#### Household income

Parent carers were asked ‘What is your household income before tax?’ and responded by selecting one option from a list (£0–£9,999, £10,000–£19,999, £20,000–£29,999, £30,000–£39,999,£40,000–£49,999,£50,000–£59,999, £60,000–£69,999, £70,000–£79,999, £80,000, or more). Given that the UK median household income is approximately £40,000 (pre‐tax), responses were recoded to create a binary variable (0 = *below‐median income*, 1 = *above median income*).

#### Young person age

Parent carers were asked ‘How old is your child (in years)?’ and selected an option of between 5 and 18 years from a drop‐down list.

#### Young person sex

Parent carers were asked ‘Is your child a…’ and selected one of the following ‘boy’, ‘girl’, or ‘other’.

#### Young person ethnicity

Parent carers were asked about their child’s ethnicity: ‘What is your child’s ethnicity?’ and selected from one of the following: Asian (Bangladeshi, Chinese, Indian, Pakistani, Asian Other), Black (Black African, Black Caribbean, Black Other), Mixed (Mixed White/Asian, Mixed White/Black African, Mixed White/Black Caribbean, Mixed Other), White British, White Non‐British (White Irish, White Gypsy/Traveller, White European, White Other), or Other (Arab, Any Other). Due to the small numbers of participants in all categories except white British, these were recoded to create a binary scale (0 = *white British* or 1 = *ethnic minority*).

#### Number of siblings

Parents were asked whether their child with SEND has any siblings. Those who responded ‘yes’ were asked three follow‐up questions: ‘how many brothers does your child with SEND have?’, ‘how many sisters does your child with SEND have?’, and ‘how many siblings does your child with SEND have that are non‐binary? Parent carers were asked to select from one of the following response options: 0, 1, 2, 3, or 4 or more. Responses to these three follow‐up questions were combined into a single variable indicating the number of siblings each young person has.

#### Young person first born

Parent carers were asked about their child’s birth order: ‘what is your child’s birth order?’ and selected from one of the following options: first‐born, second‐born, third‐born, fourth‐born, fifth‐born, or sixth‐born or later. The responses were recoded to create a binary variable indicating whether the young person was first born (0 = *no*, 1 = *yes*).

#### Young person verbal ability

To provide an indication of young people’s verbal ability, parent carers were asked ‘which of the following statements best describes your child?’. Response options were ‘can speak several words or speaks in sentences’ or ‘uses few or no words’. The responses were used to create a binary variable (0 = *verbal*, 1 = *minimally verbal*).

#### Young person educational placement

Parent carers were asked ‘What type of school does your child attend?’ and responded by selecting one of four options (0 = *mainstream school*, 1 = *special school*, 2 = *pupil referral unit*, 3 = *other*). These responses were recoded to create a binary variable (0 = *mainstream school*, 1 = *non‐mainstream*).

#### Young person education, health, and care plan

Parents/carers were asked ‘Does your child have an education, health, and care plan (EHCP)?’ and responded by selecting one of two options (0 = *no*, 1= *yes*).

#### Type of special educational need and disabilities

Given that SENDs co‐occur, parents were given a list of common SENDs and asked to select all that applied. The wording of the question was ‘what types of special educational needs or disabilities does your child have? Select all that apply’.

#### Sibling conflict

Parents were asked the following two questions about sibling conflict:

1. How often do your child’s siblings hurt or pick on them on purpose? (victimization)
2. How often does your child hurt or pick on their siblings on purpose? (perpetration)

They responded on a five‐point scale (0 = *never*, 1= *less often [than every few months]*, 2 = *every few months*, 3 = *approximately once a month*, 4 = *approximately once a week*, 5 = *most days*).

### Missing data

There were considerable missing data at each time point (T1 38%, T2 53%, T3 65%, T4 65%). These high levels of missing data were reflective of the study design and, to some extent, expected. The study design meant that there were two sources of missing data: (1) participants who took part in earlier but not later time points (i.e., sample attrition) and (2) participants who took part in later but not earlier time points (i.e., those who joined the study at the later waves of data collection). Both of these sources of missing data were captured by calculating how many time points parent carers completed questionnaires. A series of chi‐square tests were run to test whether missing data were dependent on key variables of interest. The number of time points parents took part in was not different based on the young person’s sex, χ^2^(493) = 3.57, *p* = .312, their ethnicity, χ^2^(495) = 5.16, *p* = .161, their household income, χ^2^(487) = 6.01, *p* = .111, or whether they had an EHCP, χ^2^(496) = 4.45, *p* = .217. The number of time points parent carers completed the questionnaire was dependent on educational placement. Parent carers of young people who were enrolled in a mainstream school took part at fewer time points than those in a non‐mainstream educational placement, χ^2^(496) = 10.24, *p* = .017. The statistical models that were fitted for the analyses made use of all available data such that at each time point missing data were omitted from the estimation model but cases were not deleted in a listwise manner.

### Statistical analyses

STATA/MP version 16.1 (StataCorp, 2019) was used for data analysis. Robust standard errors were calculated for all models using the sandwich estimator.

#### Research question 1

Descriptive statistics were produced to investigate the levels of sibling conflict in families with SENDs during and after the first lockdown in the United Kingdom.

#### Research question 2

To determine whether the levels of sibling conflict changed during and after the first lockdown in the United Kingdom, two multilevel mixed‐effects ordered logistic regression models were fitted (see Table S1 for details of proportional odds assumption testing). In the first model, the outcome variable was entered as sibling conflict victimization. The predictors in the fixed part of the model were the linear effect of time (i.e., whether there was an increase or decrease over time) and the quadratic effect of time (i.e., whether the *rate* of change increased or decreased). Anonymized participant number and the linear effect of time were included in the random part of the model to account for the nested structure of the data (i.e., individuals nested within time points). This model was then repeated with the outcome variable changed from sibling conflict victimization to sibling conflict perpetration.

#### Research question 3

To identify the predictors of sibling conflict, a two‐step process was implemented. In step one, twenty models were fitted (half for victimization and half for perpetration). Again, prior to fitting the models, the proportional odds assumption was tested for each of the predictors (see Table S1). Multilevel mixed‐effects ordered logistic regression models were fitted for predictors that met the proportional odds assumptions. Predictors that violated this assumption were modelled using a multilevel mixed‐effect generalized ordered logistic regression model. Each model followed a similar format to the models fitted for research question 2. For example, in the first model, the outcome variable was entered as sibling conflict victimization. The predictors in the fixed part of the model were the linear effect of time (i.e., whether there was change over time), the quadratic effect of time (i.e., whether the *rate* of change increased or decreased over time), and the young person’s sex. Anonymized participant number and the linear effect of time were included in the random part of the model. This was then repeated for nine other predictors for victimization. And then these ten models were repeated for sibling conflict perpetration.

In step two, predictors that were significant in step one were tested for multi‐collinearity. The rule of thumb approach described by O’Brien (2007) was used to assess multi‐collinearity (see Table S2). Specifically, a VIF score of above 10 or a tolerance level of .10 was assumed to violate the multi‐collinearity assumption but the context of the model was considered. For example, the variables for the linear and quadratic effect of time were expected to have high VIF scores as the quadratic effect is a function of the linear effect. All of the predictors that did not violate the multi‐collinearity assumption were then entered into two final models: one for victimization and one for perpetration. If either of the two models in step two included at least one predictor that violated the proportional odds assumption, a mixed‐effect generalized ordered logistic regression model was fitted. If all predictors met the proportional odds assumption, a multilevel mixed‐effect ordered logistic regression model was fitted.

## Results

### Sample demographics

Parent carers were asked to self‐report demographic information. Detailed sample demographics, divided by time point, are provided in Table 1 but they are described here in brief. Most of the respondents (92%) were mothers of the young person and from England (92%). Half of the sample were from a low‐income household. In terms of the young people, the mean age was approximately 11 years (range 5–18 years), most were male (70%), verbal (84%), and of white ethnicity (89%). Half the sample was enrolled in mainstream school and the other half were in a special school, pupil referral unit, or were being home‐schooled (pre‐COVID‐19 pandemic). Two‐thirds of the sample had an EHCP.

Children with a broad range of SENDs were included in the sample. Full details of the range of SENDs at each time point are provided in Table 2. Parents of approximately three‐quarters of the sample reported that their child had an autism spectrum condition. Approximately a third reported that their child had social, emotional, and mental health difficulties. A fifth reported that their child had attention deficit hyperactivity disorder. The rest of the SENDs were reported by less than a fifth of parents. There was some minor variation in the proportion of SENDs as a function of the total sample at each time point but, on the whole, these proportions were similar across time points. As expected, there was considerable co‐occurrence of SENDs across the sample. More than half of parent carers reported that their child had more than one SEND.

**Table 2:** Type of Special Educational Needs and Disabilities (SEND) as reported by the parent caregiver

	Overall, *N* (%)	Time 1, *N* (%)	Time 2, *N* (%)	Time 3, *N* (%)	Time 4, *N* (%)
Type of SEND					
Autism spectrum conditions	377 (75%)	249 (80%)	190 (80%)	124 (70%)	129 (74%)
Social, emotional, and mental health difficulties	177 (35%)	118 (38%)	94 (40%)	56 (32%)	58 (33%)
Attention deficit hyperactivity disorder	104 (21%)	68 (22%)	44 (18%)	34 (19%)	39 (22%)
Developmental language disorder	64 (13%)	49 (16%)	35 (15%)	26 (15%)	22 (13%)
Dyslexia	68 (13%)	35 (11%)	31 (13%)	21 (12%)	19 (11%)
Speech disorder or impediment	61 (12%)	39 (13%)	30 (12%)	22 (12%)	24 (14%)
Developmental coordination disorder	50 (10%)	28 (9%)	23 (10%)	20 (11%)	20 (11%)
Physical disability	39 (8%)	27 (9%)	18 (8%)	15 (8%)	10 (6%)
Attention deficit disorder	30 (6%)	21 (7%)	12 (5%)	15 (8%)	10 (6%)
Sensory processing disorder	31 (6%)	11 (4%)	10 (4%)	17 (10%)	17 (10%)
Global developmental delay	23 (5%)	14 (4%)	12 (5%)	10 (6%)	8 (5%)
Visual impairments	23 (5%)	14 (4%)	11 (5%)	7 (4%)	6 (3%)
Other^a^	60 (12%)	36 (12%)	34 (14%)	13 (7%)	13 (7%)
Co‐Occurrence of SENDs					
One SEND	202 (40%)	116 (37%)	84 (35%)	80 (45%)	74 (43%)
Two SENDs	124 (25%)	79 (25%)	62 (26%)	33 (19%)	41 (24%)
Three SENDs	96 (19%)	62 (20%)	54 (23%)	33 (19%)	31 (18%)
Four or more SENDs	82 (16%)	55 (18%)	38 (16%)	32 (17%)	28 (15%)

Note

Parents were asked to select all that applied to their child from a list.

^a^ Table only includes types of special educational needs and disabilities that were endorsed by >5% of parents (overall across all time points). The remainder were included in the other category, which includes conduct disorder, dyscalculia, Down's syndrome, epilepsy, hearing impairment, and moderate learning difficulties.

### The rates of sibling conflict during and after the first COVID‐19 lockdown

Descriptive statistics for sibling conflict, victimization (i.e., the young person with SEND being picked on or hurt by their siblings) and perpetration (i.e., the young person with SEND picking on or hurting their siblings) are shown in Table 3.

**Table 3:** Descriptive statistics for measures of parent‐report sibling conflict

	Time 1	Time 2	Time 3	Time 4
Victimisation	311 (100%)	232 (100%)	167 (100%)	174 (100%)
Never	143 (46%)	80 (34%)	44 (26%)	58 (33%)
Less often	55 (18%)	38 (16%)	35 (21%)	31 (18%)
Every few months	12 (4%)	6 (3%)	7 (4%)	6 (3%)
~Once a month	12 (4%)	14 (6%)	9 (5%)	18 (10%)
~Once a week	44 (14%)	44 (19%)	39 (23%)	29 (17%)
Most days	45 (14%)	50 (22%)	33 (20%)	32 (18%)
Perpetration	311 (100%)	232 (100%)	167 (100%)	174 (100%)
Never	70 (23%)	47 (20%)	30 (18%)	50 (29%)
Less often	45 (14%)	26 (11%)	22 (13%)	25 (14%)
Every few months	13 (4%)	8 (3%)	7 (4%)	8 (5%)
~Once a month	9 (3%)	12 (5%)	11 (7%)	14 (8%)
~Once a week	51 (16%)	53 (23%)	41 (25%)	36 (21%)
Most days	123 (40%)	86 (37%)	56 (34%)	41 (24%)

Note

Percentages represent the % of the higher‐order heading within each time point.

#### Victimization

**Figure 1:**
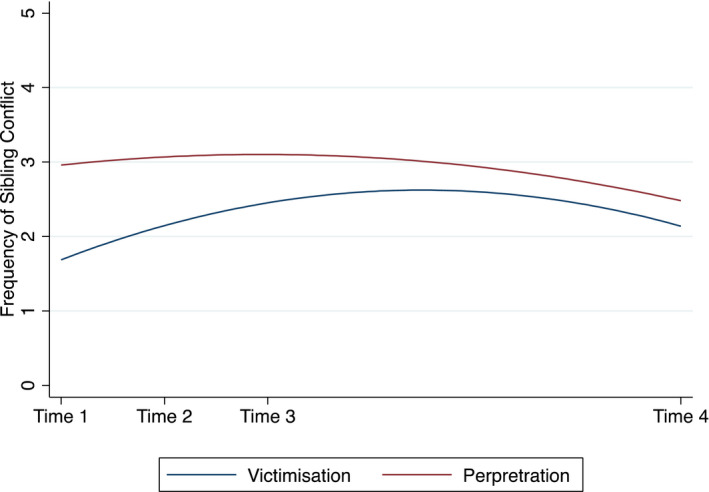
Change in sibling conflict during and after the first COVID‐19 lockdown. *Note*. The *y* axis corresponds to the response options on the questions about sibling conflict (0 = *never*, 1 = *less often [than every few months]*, 2 = *every few months*, 3 = *approximately once a month*, 4 = *approximately once a week*, 5 = *most days*).

At T1 (the beginning of the UK lockdown), over half of young people with SENDs were picked on or hurt by their siblings at least once (i.e., any response other than ‘never’). At T2, the victimization rate increased to two thirds and reached nearly three‐quarters of the sample by the T3. However, at T4 (i.e., when schools fully re‐opened for face‐to‐face teaching), there appeared to be a slight decrease in the rate of victimization of young people with SENDs. These observations were corroborated by the multi‐level ordered logistic regression model (Table 4, Model 1). As shown in Figure 1, there was an initial increase in sibling conflict (as demonstrated by the linear effect of time) but this decreased after lockdown (quadratic effect of time). Therefore, young people with SENDs were more likely to be victimized by their siblings as lockdown progressed and schools remained closed for face‐to‐face teaching for most students; the victimization rate started to decline when schools fully reopened for face‐to‐face teaching.

**Table 4:** Predictors of sibling conflict during and after lockdown

Predictor	Victimization			Perpetration		
	Model	Odds ratios [95% confidence intervals]	*p* Value	Model	Odds ratios [95% confidence intervals]	*p* Value
Linear effect of time	1	2.23 [1.72, 3.25]	<.001	2	1.25 [0.90, 1.73]	.176
Quadratic effect of time	1	0.89 [0.85, 0.93]	<.001	2	0.95 [0.91, 1.00]	.**045**
Age	3	1.03 [0.95, 1.12]	.451	13	0.90 [0.82, 0.98]	.**012**
Boy	4	1.31 [0.67, 2.58]	.434	14	0.94 [0.47, 1.86]	.850
First born	5	2.18 [1.15, 4.11]	.**017**	15	2.15 [1.12, 4.13]	.**022**
Number of siblings	6	1.44 [1.09, 1.90]	.**009**	16	1.37 [1.00, 1.87]	.**048**
Autism spectrum conditions	7	0.82 [0.42, 1.63]	.581	17	1.21 [0.58, 2.52]	.610
Attention deficit hyperactivity disorder	8	2.02 [0.98, 4.17]	.057	18	4.48 [2.08, 9.63]	**<.001**
Social, emotional, and mental health difficulties	9	1.39 [0.74, 2.62]	.306	19	1.32 [0.68, 2.58]	.408
Minimally verbal	10	0.13 [0.05, 0.30]	**<.001**	20	0.29 [0.12, 0.67]	.**004**
Non‐mainstream educational placement	11	0.31 [0.16, 0.58]	**<.001**	21	0.44 [0.24, 0.83]	.**011**
Education, health, and care plan	12	0.33 [0.17, 0.64]	.**001**	22	0.52 [0.27, 0.99]	.**048**

#### Perpetration

At T1, over three‐quarters of young people with SENDs picked on or hurt their siblings (i.e., any response other than ‘never’). At T2 and T3, the equivalent figure was approximately four out of five young people. As with victimization, there appeared to be a slight decrease in perpetration rates at T4 when just over two‐thirds of young people with SENDs pick on or hurt by their siblings. The multi‐level ordered logistic regression model (Table 4, Model 2) showed that there was no significant change in sibling conflict perpetration over time (the linear effect of time was not significant). The decrease at T4 was significant (the quadratic effect of time was significant). Therefore, young people with SENDs were not more or less likely to pick on or hurt their siblings as lockdown progressed but there was a small decrease after lockdown eased and schools fully re‐opened for face‐to‐face teaching.

### Predictors of sibling conflict during and after the first COVID‐19 lockdown

A series of individual models were fitted to investigate which factors predicted sibling conflict during and after the first COVID‐19 lockdown (Table 4, Models 3–22).

#### Victimization

A number of predictors of sibling conflict victimization were identified (Table 4, Models 3–12). Birth order and the total number of siblings predicted higher levels of sibling conflict victimization. First‐born young people were more likely to be victimized by their siblings compared with those who were born second or later. Additionally, as the number of siblings increased so did the frequency of victimization. Verbal ability, educational placement, and having an EHCP were predictors of lower levels of sibling conflict victimization. Young people who were minimally verbal, enrolled in non‐mainstream educational placement, or had an EHCP were less likely to be victimized by their siblings compared with those who were verbal, enrolled in a mainstream school, or those who did not have an EHCP, respectively.

All of the predictors that were significant in the previous step were entered into a final model of sibling conflict victimization. That is, all of the significant predictors of sibling conflict victimization (Table 4, Models 1,5–6, and 10–12) were entered into a single sibling conflict victimization model (Table 5, Model 1). This allowed for the investigation of whether the predictors remained significant after accounting for the variance explained by all other significant predictors. The only predictor that was no longer significant in the final model was educational placement. Therefore, in the final model, young person age, birth order, number of siblings, verbal ability, and whether they had an EHCP were significant predictors of sibling conflict victimization.

**Table 5:** Final models of siblings conflict

Predictor	Model 1: Victimization		Model 2: Perpetration	
	Odds ratios [95% confidence intervals]	*p* Value	Odds ratios [95% confidence intervals]	*p* Value
Linear effect of time	2.28 [1.65, 3.16]	<.001	1.36 [0.97, 1.89]	.072
Quadratic effect of time	0.90 [0.85, 0.94]	<.001	0.94 [0.90, 0.99]	.020
Age	–	–	0.87 [0.80, 0.95]	.001
First born	2.93 [1.53, 5.61]	.001	2.51 [1.28, 4.90]	.007
Number of siblings	1.67 [1.25, 2.23]	<.001	1.58 [1.14, 2.20]	.007
Attention deficit hyperactivity disorder	–	–	4.06 [1.87, 8.81]	<.001
Minimally verbal	0.21 [0.08, 0.51]	.001	0.33 [0.12, 0.85]	.022
Non‐mainstream educational placement	0.65 [0.33, 1.29]	.217	0.82 [0.39, 1.73]	.611
Education, health, and care plan	0.48 [0.24, 0.95]	.036	0.61 [0.29, 1.28]	.191

#### Perpetration

Sibling conflict perpetration was also significantly predicted by many factors (Table 4, Models 13–22). As with victimization, birth order, and the total number of siblings were associated with higher levels of sibling conflict perpetration. First‐borns and those with more siblings were more likely to victimize their siblings compared with second or later‐born or those with fewer siblings, respectively. In addition to this, unlike sibling conflict victimization, those with attention deficit hyperactivity disorder were more likely to pick on or hurt their siblings compared with those without attention deficit hyperactivity disorder. As with sibling conflict victimization, verbal ability, educational placement, and whether the young person had an EHCP were associated with lower levels of sibling conflict perpetration. Young people who were minimally verbal, enrolled in non‐mainstream educational provision, and had an EHCP were less likely to pick on or hurt their siblings compared with those who were verbal, were enrolled in mainstream education, or those without an EHCP, respectively. In addition to this, unlike sibling conflict victimization, age was a significant predictor of sibling conflict perpetration. Older children were less likely to pick on or hurt their siblings compared to younger children.

All of the predictors that were significant in the previous step were entered into a final model of sibling conflict victimization. That is, all of the significant predictors of sibling conflict perpetration (Table 4, Models 2, 13, 15–16, 18, and 20–22) were entered into a single sibling conflict perpetration model (Table 5, Model 2). Two predictors were no longer significant in this model, educational placement and whether the young person had an EHCP. Therefore, young person age, birth order, number of siblings, whether they had attention deficit hyperactivity disorder, and their verbal ability were significant predictors of sibling conflict perpetration during and after the first COVID‐19 lockdown in the United Kingdom.

## Discussion

This longitudinal study investigated sibling conflict during and after the first COVID‐19 lockdown in the United Kingdom in families with SENDs. The frequency with which young people with SENDs were picked on or hurt by their siblings increased during the first lockdown in the United Kingdom. On the other hand, the frequency with which young people with SENDs picked on or hurt their siblings remained mostly stable during the lockdown. A few months after the first lockdown, once schools had fully re‐opened for face‐to‐face teaching, the frequency of victimization and perpetration decreased but did not reach the same levels as the first month of lockdown, suggesting that the rates of conflict had not yet stabilized. Those who were minimally verbal appeared to be somewhat protected from sibling conflict, both in terms of victimization and perpetration.

The rates of sibling conflict during the first COVID‐19 lockdown in the United Kingdom were high. At their highest level (the third month of lockdown), three out of four of young people with SENDs were being picked on or hurt by their siblings and four out of five were picking on or hurting their siblings on purpose (according to parent carer reports). At the extreme end, one in five young people were being picked on or hurt by their siblings on most days and one in three picked on or hurt their siblings on most days. These findings have implications for future pandemic‐related lockdowns. Spending extended periods of time at home appears to have a negative effect on sibling relationships in families with SENDs. This is not unexpected – spending more time together in close proximity provides more opportunity for conflict to arise. This is supported by the finding that once schools had fully re‐opened for face‐to‐face teaching, sibling conflict rates began to decrease. The scale of the problem, however, should be cause for alarm given that persistent sibling conflict is associated with poor outcomes in the short and long term (Bowes et al., 2014; Dantchev et al., 2018, 2019; Dantchev & Wolke, 2018; Liu et al., 2020; Lopes et al., 2019; Tucker, Finkelhor, Turner, et al., 2013; van Berkel et al., 2018), specifically in young people with SENDs (Toseeb, McChesney, Oldfield, et al., 2020; Toseeb et al., 2018).

Whilst direct comparisons were not made to neurotypical families, it is likely that the effects observed were more pronounced for families of young people with SENDs. These families are much more reliant on support from specialist agencies on a day‐to‐day basis and the rates of social and mental health difficulties tend to be higher in neurotypical family members (Constantino et al., 2006; Hoffman et al., 2009). Previous work with this sample has demonstrated that parents felt overwhelmed by having to deal with their child with SENDs all day every day without support and that they suffered psychological distress as a result (Asbury et al., 2021; Toseeb, Asbury, et al., 2020). Taken together, the findings appear to be in line with previous work suggesting that increases in parental distress during the COVID‐19 pandemic are associated with increases in conflict within the family (Lee & Ward, 2020).

Young people with the most severe or complex needs were somewhat protected from sibling conflict during and after the COVID‐19 lockdown in the United Kingdom. Those who were minimally verbal were less likely to be involved in sibling conflict as perpetrators and victims. Additionally, those who had an EHCP were less likely to be victimized by siblings. It may be that siblings of young people with complex or severe SENDs perceive the attention directed towards their affected sibling as warranted and therefore are less likely to compete for parental resources (Kowal, Krull, Kramer, & Crick, 2002). Alternatively, it may be that siblings of those with complex or severe SENDs adopt a more parent‐like approach in the face of adversity. This is in line with the family systems approach whereby if one member of the family is affected with a SEND, then other members of the family tend to adapt to accommodate (Turnbull, Summers, & Brotherson, 1986). This novel finding warrants further investigation in future research to explore some of the reasons why young people with complex or severe needs are somewhat protected from sibling conflict.

The findings also highlight the need to consider siblings of young people with SENDs. Consistently high proportions of the siblings of young people with SENDs were being hurt or picked on by the young person with SENDs on a regular basis during the lockdown. Given that persistent sibling conflict is associated with poorer mental health, it is likely that siblings of young people with SENDs also suffered increased levels of psychological distress during the lockdown. Future work should consider the effect of the COVID‐19 lockdowns on the mental health of siblings of young people with SENDs. Such work is likely to inform support targeted at SENDs families considering the unique needs of parents, the young person with SENDs, and their siblings.

There are a number of strengths of the current study. To the best of the author’s knowledge, it is the only study to investigate sibling conflict during the COVID‐19 pandemic in the United Kingdom. Data were collected from the first day of the first UK lockdown and, where possible, families with followed up at multiple points to investigate the change in sibling conflict. A number of drawbacks should be borne in mind when interpreting the findings. Firstly, missing data was dependent on educational placement. Secondly, a high proportion of the sample had an autism spectrum condition, which may affect generalizability to other SENDs. The effects were compared for those with and without autism spectrum conditions and there were no significant effects but this may be due to the low numbers of those without autism spectrum conditions. Thirdly, parent carer reports of sibling conflict were used. This may be problematic because parents are not always aware of the conflict between siblings. Self‐report may also be problematic because young people with SENDs may not recognize harmful behaviour. Future research on families with SENDs should adopt a multi‐informant perspective to allow for a comprehensive account of sibling conflict. Finally, an opportunity sample was used. This meant that families who were struggling the most may not have had the time to take part in an online survey about their experiences. The findings presented here should be combined with evidence from other sources such as routinely collected administrative data and or data from population cohort studies. This triangulating of evidence from multiple sources will help to address some of the drawbacks associated with online surveys making use of opportunity samples.

These findings shed new light on sibling conflict during and after the first lockdown in the United Kingdom. They provide the first set of evidence of the negative effects of lockdown on sibling relationships in families of young people with SENDs. Future work should consider the longer term implications of the lockdown for families in which one or more child has SENDs in larger and more representative samples. The study highlights the need to consider siblings and their relationships in planning support for families with SENDs during future pandemic‐related lockdowns.

## Conflicts of interest

All authors declare no conflict of interest.

## Author contribution

**Umar Toseeb:** Conceptualization (equal); Data curation (equal); Formal analysis (equal); Funding acquisition (equal); Investigation (equal); Methodology (equal); Project administration (equal); Visualization (equal); Writing – original draft (equal); Writing – review & editing (equal).

## Supporting information


**Table S1**. Proportional odds assumption testing.
**Table S2**. Multi‐collinearity metrics for variables in final models.

## Data Availability

Data collection for this study is ongoing and so it is not possible to make the data available. Once data collection is complete, anonymized data will be made available in an open‐access repository.
