# Supplementary material for: Sibling conflict during COVID‐19 in families with special educational needs and disabilities
Source: Br J Educ Psychol. 2021 Aug 22;92(1):e12451. doi: 10.1111/bjep.12451 (PMC8646725; doi:10.1111/bjep.12451)
Supplement: Supplementary file 1 — Table S1. Proportional odds assumption testing. Table S2. Multi‐collinearity metrics for variables in final models. [file BJEP-92-319-s001.docx]

**Table S1.**

*Proportional Odds Assumption Testing*

|  | **Chi-squared value** | **P value** |
| --- | --- | --- |
| **Sibling Conflict Victimisation** |  |  |
| Linear effect of time | 1.88 | .758 |
| Quadratic effect of time | 1.76 | .780 |
| Age | 14.23 | .007 |
| Sex | 5.50 | .240 |
| First born | 1.26 | .868 |
| Number of siblings | 15.50 | .004 |
| Autism spectrum conditions | 1.29 | .864 |
| Attention deficit hyperactivity disorder | 6.21 | .184 |
| Social, emotional, and mental health difficulties | 1.58 | .812 |
| Verbal ability | 9.17 | .057 |
| Educational placement | 1.44 | .838 |
| Education, health, and care plan | 5.48 | .241 |
| **Sibling Conflict Perpetration** |  |  |
| Linear effect of time | 8.37 | .079 |
| Quadratic effect of time | 5.94 | .204 |
| Age | 4.40 | .354 |
| Boy | 2.86 | .581 |
| First born | 3.11 | .540 |
| Number of siblings | 9.23 | .056 |
| Autism spectrum conditions | 5.81 | .214 |
| Attention deficit hyperactivity disorder | 2.37 | .668 |
| Social, emotional, and mental health difficulties | 4.42 | .352 |
| Minimally-verbal | 6.24 | .182 |
| Special school | 1.21 | .876 |
| Education, health, and care plan | 5.23 | .264 |

**Table S2.**

*Multi-collinearity Metrics for Variables in Final Models*

|  | VIF | Tolerance |
| --- | --- | --- |
| **Model 1 - Victimisation** |  |  |
| Linear effect of time | 18.25 | .05 |
| Quadratic effect of time | 18.19 | .05 |
| First born | 1.12 | .89 |
| Number of siblings | 1.14 | .88 |
| Minimally verbal | 1.13 | .88 |
| Non-mainstream educational placement | 1.37 | .73 |
| Education, health, and care plan | 1.30 | .77 |
| **Model 2 – Perpetration** |  |  |
| Linear effect of time | 18.43 | .05 |
| Quadratic effect of time | 18.30 | .05 |
| Age | 1.09 | .91 |
| First born | 1.12 | .89 |
| Number of siblings | 1.16 | .86 |
| Attention deficit hyperactivity disorder | 1.03 | .97 |
| Minimally verbal | 1.19 | .84 |
| Non-mainstream educational placement | 1.42 | .71 |
| Education, health, and care plan | 1.32 | .76 |
